# Climate and landscape drivers of tree decline in a Mediterranean ecoregion

**DOI:** 10.1002/ece3.437

**Published:** 2013-01-10

**Authors:** Niels C Brouwers, Jack Mercer, Tom Lyons, Pieter Poot, Erik Veneklaas, Giles Hardy

**Affiliations:** 1 State Centre of Excellence for Climate Change, Woodland and Forest Health, School of Environmental Science, Murdoch University90 South Street, Murdoch, Western Australia, 6150, Australia; 2 Marlak Environmental ServicesAlbany, Western Australia, 6331, Australia; 3 State Centre of Excellence for Climate Change, Woodland and Forest Health, School of Plant Biology, University of Western Australia (M084)35 Stirling Highway, Crawley, Western Australia, 6009, Australia; 4 State Centre of Excellence for Climate Change, Woodland and Forest Health, School of Biological Sciences and Biotechnology, Murdoch University90 South Street, Murdoch, Western Australia, 6150, Australia

**Keywords:** Climate change, die-off, dieback, *Eucalyptus wandoo*, forest canopy health, fragmentation, southwest Western Australia, tree crown health

## Abstract

Climate change and anthropogenic land use are increasingly affecting the resilience of natural ecosystems. In Mediterranean ecoregions, forests and woodlands have shown progressive declines in health. This study focuses on the decline of an endemic woodland tree species, *Eucalyptus wandoo* (wandoo), occurring in the biodiversity hotspot of southwest Western Australia. We determined the change in health of wandoo stands between 2002 and 2008 across its geographic and climatic range, and associated this change in health with non-biotic variables focusing on: (1) fragment metrics; (2) topography; (3) soil characteristics; and (4) climate. Only fragment metrics and climate variables were found to be significantly related to the observed change in health. Stands that were small with high perimeter/area ratios were found to be most sensitive to health declines. Recent increases in autumn temperatures and decreases in annual rainfall were negatively affecting health of wandoo most prominently in the low rainfall zone of its climatic range. Together, these results suggest the onset of range contraction for this ecologically important species, which is likely to be exacerbated by projected future changes in climate. Our results emphasize the importance of establishing monitoring programs to identify changes in health and decline trends early to inform management strategies, particularly in the sensitive Mediterranean ecoregions.

## Introduction

Climate change, habitat loss and fragmentation are important drivers of biodiversity decline around the world (Mantyka-Pringle et al. 2012). Forest and woodland ecosystems are increasingly showing the effects of these change processes (van Mantgem and Stephenson 2007; van Mantgem et al. 2009; Phillips et al. 2009; Allen et al. 2010; Barbeta et al. 2011; Carnicer et al. 2011; Peng et al. 2011; Huang and Anderegg 2012). Many dominant tree species have shown distinct periods of dieback and mortality linked to frequent short-term extreme weather events (i.e. droughts and heatwaves) (Phillips et al. 2009; Allen et al. 2010; Huang and Anderegg 2012; Matusick et al. 2012), or shown gradual increases in mortality rates and/or reduced growth rates linked to the long-term global increases in temperature and changes in rainfall (Jump et al. 2006; van Mantgem and Stephenson 2007; Sarris et al. 2007, 2011; van Mantgem et al. 2009; Dulamsuren et al. 2010; Carnicer et al. 2011; Peng et al. 2011; Vilà-Cabrera et al. 2011). As many of the changes in climate are projected to persist or intensify (IPCC 2007a,b), future declines and related change processes in forested ecosystems are likely to become more prevalent (IPCC 2007a; Phillips et al. 2009; Allen et al. 2010; Peng et al. 2011).

Projected global climate change trends are likely to have different effects on ecosystems and individual species (Hansen et al. 2001; Allen et al. 2010). In several studies around the world, co-occurring tree species were found to respond differently to drought and heating events (Allen et al. 2010). For example, coniferous and deciduous species showed varying mortality rates in response to recurring droughts in mixed stands across Spain (Peñuelas et al. 2001). A recent study in southwest Western Australia revealed differences in dieback responses in co-occurring *Eucalyptus* species after an extreme drought and heating event (Matusick et al. 2012). These findings indicate that species demonstrate different levels of resilience to changes in climate, which will likely result in shifts in species composition, range, and ecosystem functioning (Hansen et al. 2001). Under the current climate change projections, it is expected that range shifts will occur for eucalypts (Hughes et al. 1996) and other woody species particularly at the boundaries of their current range (Jump et al. 2009). However, the assumed species responses to changes in climate are difficult to measure due to the complexity and long temporal scales of the processes involved (Jump et al. 2009). In this study, we document the changes in health of an endemic southwest Australian tree species over a 6-year period across its range and associate it with the changes in the local climate.

The southwest of Western Australia (SWWA) is a unique ecoregion, and one of five globally recognized “biodiversity hotspots” with a Mediterranean climate (Klausmeyer and Shaw 2009; Mittermeier et al. 2011). Like most other Mediterranean ecoregions, SWWA has undergone extensive clearing of native vegetation (∼70%) resulting in a highly fragmented landscape (Beard 1990; Shepherd et al. 2002). Changes in climate in this region have especially been pronounced since the mid-1970s, with mean annual temperatures increasing 0.45°C and annual rainfall decreasing by 14% (Bates et al. 2008). This drying and warming trend is projected to continue with estimates of up to 40% rainfall reduction and mean annual temperature increases of up to 5°C by 2070 (CSIRO & BOM 2007). In many Mediterranean ecoregions, similar climatic trends have been observed and projected (IPCC 2007b), indicating the significance of these change processes and their potential impacts on biota inhabiting these ecoregions (Klausmeyer and Shaw 2009).

Based on the projected changes in climate, large parts of Mediterranean ecoregions and specifically SWWA are projected to become increasingly unsuitable for the species they currently support (Hughes et al. 1996; Klausmeyer and Shaw 2009; Laurance et al. 2011). Over the last 30 years, several dominant tree species endemic to SWWA have declined in health, which has been considered to be related to the gradual changes in climate (Hooper and Sivasithamparam 2005; Cai et al. 2010), extreme weather events (Brouwers et al. 2012), and fragmentation effects (Mercer 2003, 2008). Similar declines in health and growth of dominant tree species have been related to climatic changes in other Mediterranean ecoregions (e.g. Jump et al. 2006; Allen et al. 2010; Barbeta et al. 2011; Carnicer et al. 2011; Sarris et al. 2011; Sánchez-Salguero et al. 2012). None of these Mediterranean studies, however, investigated the possible drivers of the observed declines across the entire geographic and climatic range of a species. This study investigates the role of climate and landscape variables on changes in tree health, and provides a first test of how the range of a SWWA tree species might shift with climate change as suggested in modeling studies (Hughes et al. 1996; Klausmeyer and Shaw 2009).

In apparent parallel with the commencement of decreases in annual rainfall and increases in mean annual temperature since the mid-1970s in SWWA (Bates et al. 2008), the tree species *Eucalyptus wandoo* Blakely subsp. *wandoo* (wandoo) has shown signs of decline across its range (Hooper and Sivasithamparam 2005; Gaynor 2008). Subsequent public concern led to the funding of a variety of studies to elucidate the causal factors for the observed declines (Wandoo Recovery Group 2006). As a part of this effort, we performed a landscape-scale assessment investigating the relationships between the change in wandoo canopy health assessed over a 6-year period and variables focussing on: (1) fragment metrics; (2) topography; (3) soil characteristics; and (4) climate.

## Materials and Methods

### Study species and area

**Figure 1:**
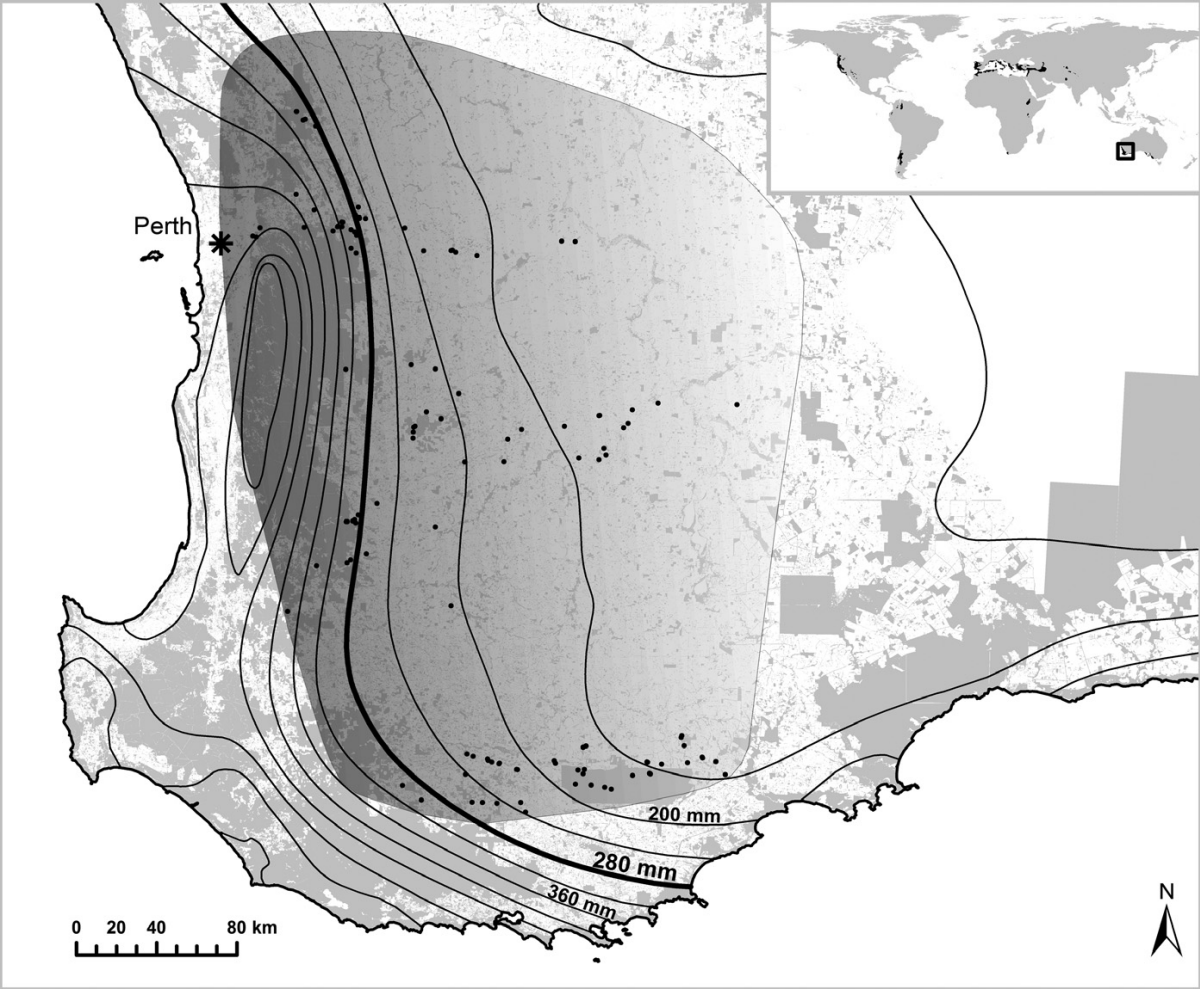
The climatic and geographic range of wandoo across the southwest of Western Australia. The world map indicates Mediterranean climate regions, with black highlights representing Köppen climate symbols Csa and Csb following Peel et al. (2007), and the black square indicating the study region in Australia. Shaded central outline represents the wandoo range based on the point records from this study and the NatureMap database (DEC 2012). Black dots indicate the survey plots used in this study. Underlying gray fields indicate the remaining native vegetation cover in the region, with white indicating areas of human land use, predominantly agriculture. Lines indicate the 30-year average winter rainfall 40 mm stepped isohyets based on the annual rainfall data from 1976 to 2005. The bold 280 mm isohyet indicates the *x*-axis intercept of the linear relationship between winter rainfall and crown health change shown in Fig. 3b. Across its range, wandoo crown health was found to increasingly decline between 2002 and 2008 from higher (dark, left) to lower (light, right) rainfall zones.

The Mediterranean climate of the SWWA is characterized by warm to hot, dry summers and mild-to-cool, wet winters (following Peel et al. 2007). Most (∼80%) rainfall falls between April and October (Bates et al. 2008) and a distinct seasonal dry period occurs between October and April lasting between 4 and 8 months (Beard 1990). Wandoo has a broad range across SWWA (between Latitude 31°0 and 34°30′S and Longitude 115°50 and 118°55′E; Fig. 1), occurring in areas receiving between ∼300 and 1000 mm annual rainfall and experiencing average temperatures between ∼5°C in winter and ∼34°C in summer (derived from Australian Water Availability Project (AWAP) dataset, see Jones et al. 2009; Raupach et al. 2009, 2011).

Wandoo commonly occurs across what is locally known as the northern and southern jarrah (*Eucalyptus marginata*) forest region in the west, and extends into the wheatbelt region toward the east (Beard 1990). Native vegetation in these three regions has progressively been cleared for agriculture, mining and other human land uses with ∼60% cleared in the jarrah forest and ∼90% in the wheatbelt region (Beard 1990; Shepherd et al. 2002) (Fig. 1). Predominantly, wandoo occurs on a variety of loamy soil types over clay subsoils, and is associated with *E. accedens* (powderbark wandoo), *Corymbia calophylla* (marri), *E. marginata* (jarrah) and *Allocasuarina huegeliana* (rock sheoak) in the west, and with *E. loxophleba* (York gum) and *E. salmonophloia* (salmon gum) in the east (Beard 1990). Specific wheatbelt associations are with *E. falcata* (silver mallet), *E. gardneri* (blue mallet), *E. astringens* (brown mallet), *E. occidentalis* (flat-topped yate) and *E. rudis* (flooded gum). Wandoo mainly occurs as open woodland characterized by less than 40% overstory crown cover, with generally sparse understory vegetation (Beard 1990).

Wandoo trees and woodlands have a high conservation value, as they provide a variety of products and services such as honey, timber, watershed protection, and recreation, as well as supporting high levels of biodiversity by providing a variety of habitats (Majer and Recher 1988; Beard 1990; Majer et al. 2003; Wandoo Recovery Group 2006). Compared with other valued endemic *Eucalyptus* species in the SWWA, wandoo supports significantly more invertebrate fauna (Majer and Recher 1988; Majer et al. 2003). This abundant invertebrate resource supports a diverse woodland fauna (Cousin and Phillips 2008), illustrating the importance of retaining wandoo woodlands for biodiversity conservation in SWWA.

### Field surveys

In response to declining health observed for wandoo in early 2000 (Hooper and Sivasithamparam 2005; Wandoo Recovery Group 2006), surveys were conducted in 2002 and again in 2008 both between the 26th of March and the 11th of June (Mercer 2003, 2008). In the surveys, health on 126 wandoo-dominated plots was assessed along three transects traversing the broad climatic range of the species (Fig. 1). Lacking accurate distribution maps for wandoo, potential locations were identified using local expert knowledge as well as opportunistic searches along main east-west-oriented roads and secondary routes connecting north and south. Plots were selected based on the following criteria: (1) wandoo canopy dominance of the plot had to be >90%; (2) the plot and surrounding area had to show no clear signs of waterlogging; and (3) had to be accessible via roads and tracks.

Each plot was 2500 m^2^, with the majority of plots measuring 50 × 50 m (*n* = 111) and oriented in a north–south direction, whereas an additional 15 plots measured 25 × 100 m and ran parallel to streams or roads with varying directions. The plots were used to represent each landscape location. For each plot, a GPS point was taken on the northeast corner using a handheld Garmin 12 (GARMIN International, Kansas) with an accuracy of 15 m.

The majority of plots showed high variability in crown health between individual wandoo trees. In order to capture the overall canopy health, all the trees in the survey plots were used to assess the average crown health of wandoo following an adaptation of the established and widely used Grimes (1978) assessment method described by Abbott (1992). During four field trials, the Abbott (1992) method was found most accurate in capturing wandoo crown health in comparison with the health assessment techniques described by Grimes (1978) and Mercer (#b^500^). Average crown health of wandoo was assessed by estimating and adding measures of: (1) overall leaf density (range: 0–9, i.e. leaves absent–dense); (2) incidence of dead branches (0–9, all dead–no dead); and (3) the contribution of epicormics (i.e. shoots that develop from dormant buds following stress) to crown and bole (0–6, tree dead–no epicormics present) resulting in a crown health rating ranging from 0 to 24, i.e. dead–healthy (Abbott 1992; Mercer 2003, 2008). To be able to assign a health rating representing the average condition for all wandoo trees together, the surveyor walked around the perimeter and in two diagonals through each plot, making a group assessment of trees falling in three individual diameter classes (5–20, 20–40 and >40 cm), to estimate the average value of crown health for the whole plot as described above. Additionally, a descriptive assessment was made recording details on the average tree density per plot, diameter class, the surrounding land use, and evidence of recent fire damage (see further Mercer 2008). To ensure continuity and accuracy between successive health ratings, the same assessor carried out the 2002 and 2008 assessments. The 6-year period between surveys made it possible to estimate the change in wandoo crown health at each survey plot.

The crown health ratings measured in 2002 and 2008 at each plot were normalized and the difference between ratings (2008 minus 2002) used to represent the magnitude of crown health change over time (index range: −1/0/+1; i.e. maximum decline/no change/maximum improvement). This index was used to associate health change with variables extracted and computed from readily available spatial datasets for the SWWA (Table 1). The GPS points of the plots were used to extract these data using MATLAB 7.7.0 (R2008b, MathWorks, Massachusetts), and ArcGIS 10 (ESRI, California). Eleven plots were excluded where health ratings were affected by fires that occurred before and between the two surveys, resulting in a total sample of *n* = 115. We associated health changes observed in the plots with (1) fragment metrics (area, perimeter, and the shape complexity index: Fractal dimension (FRAC) = 2*ln(perimeter)/ln(area)); (2) topographic variables (height, slope, aspect); (3) soil characteristics (shallowness, salinity; following Harper et al. (2005)); and (4) climate-related variables including rainfall, temperature and soil moisture estimates, and the changes in these variables between the two surveys calculated as annual and seasonal means (Table 1). The fragments used in the fragment metric analyses were individual digitized outlines indicating native vegetation remnants (see dataset Table 1). In the comparison with the health change measured in the plots, only the fragments that included a survey plot were used. The potential influence of the soil characteristics on wandoo health change was calculated for each plot as a percentage using the soil properties (i.e. soil substrate and texture) derived from the digital soil map available for SWWA (see Harper et al. 2005 and Table 1). For soil shallowness (i.e. likelihood of presence of shallow soil profile i.e. <2 m to rock layer) and salinity (i.e. likelihood of presence of salinity sensitive soils), 0% indicated a low influence, and 100% a potential high (negative) influence on wandoo at the individual plots (Harper et al. 2005). The climate-related variables were generated from the AWAP dataset (Raupach et al. 2009, 2011), which includes gridded data surfaces (5 × 5 km) for rainfall and temperature based on data recorded by the entire weather stations network managed by the Australian Bureau of Meteorology, and grids for modeled soil moisture as calculated by the AWAP consortium. This was the best consistent climate dataset that was freely available for the SWWA. For more details on these datasets, see Raupach et al. (2009, 2011) and Jones et al. (2009).

**Table 1:** Data and variables that were used in the landscape-scale assessment of wandoo health change across its geographic range in southwest Western Australia (SWWA)

Data description	Variable computed for the analyses	Units/Categories	Details of parent dataset used
Fragment metrics	Area	Square meters (m^2^)	Native vegetation current extent, 2010, DAF, WA, 10 m res
	Perimeter	Meters (m)	Native vegetation current extent, 2010, DAF, WA, 10 m res
	Fractal dimension (FRAC)	Fraction (1–2)	Native vegetation current extent, 2010, DAF, WA, 10 m res
Topographic position	Elevation	Meters (m)	Digital Elevation Model, South West basins, 2008, Landgate/CSIRO, WA, 10 m res
	Slope	Degrees (°)	Digital Elevation Model, South West basins, 2008, Landgate/CSIRO, WA, 10 m res
	Aspect	North or South facing	Digital Elevation Model, South West basins, 2008, Landgate/CSIRO, WA, 10 m res
Soil characteristics	Shallowness	Percentage (%)	Soil-landscape mapping South-Western Australia, 2008, DAF, WA, 1.5 km res
	Salinity	Percentage (%)	Soil-landscape mapping South-Western Australia, 2008, DAF, WA, 1.5 km res
Climate-related variables	Av rainfall (1976–2005)	Millimeters (mm)	Australian Water Availability Project (AWAP), Run 26c, 2011, CSIRO, 5 km res
Calculated per:	Av temperature (1976–2005)	Degree Celsius (°C)	Australian Water Availability Project (AWAP), Run 26c, 2011, CSIRO, 5 km res
Annum,	Av minimum temp (1976–2005)	Degree Celsius (°C)	Australian Water Availability Project (AWAP), Run 26c, 2011, CSIRO, 5 km res
Summer (Dec, Jan, Feb),	Av maximum temp (1976–2005)	Degree Celsius (°C)	Australian Water Availability Project (AWAP), Run 26c, 2011, CSIRO, 5 km res
Autumn (Mar, Apr, May),	Av soil moisture (1976–2005) L1	Fraction (0–1)	Australian Water Availability Project (AWAP), Run 26c, 2011, CSIRO, 5 km res
Winter (Jun, Jul, Aug),	Av soil moisture (1976–2005) L2	Fraction (0–1)	Australian Water Availability Project (AWAP), Run 26c, 2011, CSIRO, 5 km res
Spring (Sep, Oct, Nov)	Ch rainfall (av 2008–av 2002)	Millimeters (mm)	Australian Water Availability Project (AWAP), Run 26c, 2011, CSIRO, 5 km res
	Ch temp (av 2008–av 2002)	Degree Celsius (°C)	Australian Water Availability Project (AWAP), Run 26c, 2011, CSIRO, 5 km res
	Ch min temp (av 2008–av 2002)	Degree Celsius (°C)	Australian Water Availability Project (AWAP), Run 26c, 2011, CSIRO, 5 km res
	Ch max temp (av 2008–av 2002)	Degree Celsius (°C)	Australian Water Availability Project (AWAP), Run 26c, 2011, CSIRO, 5 km res
	Ch soil moist (av 2008–av 2002) L1	Fraction (0–1)	Australian Water Availability Project (AWAP), Run 26c, 2011, CSIRO, 5 km res
	Ch soil moist (av 2008–av 2002) L2	Fraction (0–1)	Australian Water Availability Project (AWAP), Run 26c, 2011, CSIRO, 5 km res

All parent datasets included spatial data for the whole of SWWA. The 12 climate-related variables listed were calculated as annual and seasonal averages totalling 5 × 12 variables that were included in the analysis. DAF: Department of Agriculture and Food; DEC: Department of Environment and Conservation; CSIRO: Commonwealth Scientific and Industrial Research Organisation; for details on AWAP datasets, see http://www.csiro.au/awap; WA: Western Australia; res: maximum resolution (or coarseness) of the dataset; Ch av 2008–av 2002: Change variable as the difference between the 6-year average for 2008 and 2002 (i.e. av 2002–2007 minus av 1996–2001); L1: Upper soil layer up to 0.2 m deep; L2: Lower soil layer between 0.2 and 1.5 m deep.

To smooth the influence of the monthly interpolation errors in the original meteorological gridded datasets (Jones et al. 2009), we calculated 30-year and 6-year average values for the individual climate-related variables per annum (i.e. 12 months) and season (i.e. summer [December, January, February], autumn [March, April, May], winter [June, July, August], spring [September, October, November]) (Table 1). The 30-year long-term average for all climate variables was based on the period from 1976 to 2005. To generate variables representing the relative changes in climate and smoothing out annual variability, the difference between the 6-year average before (1996–2001, i.e. av 2002) and between (2002 and 2007, i.e. av 2008) the surveys was used. Thus, for each climate-related variable, a “change” variable was generated by subtracting av 2008–av 2002 (see Table 1). The resulting climate change variables were used in finding relationships with the wandoo health change variable.

### Statistical analyses

All analyses were performed using R (2.12.0, http://www.r-project.org) and following steps described in Logan (2010). Where necessary, continuous variables (Table 1) were transformed or outliers removed to meet the assumptions of normality. To investigate the relationships between the continuous fragment metrics and wandoo health change, we performed linear regression analysis. To investigate the relationships between the continuous topographic variables and wandoo health change, linear regression analysis was performed. The relationship between aspect (North or South) and wandoo canopy health change was analyzed using a Welch two-sample *t*-test. To investigate the relationships between the continuous soil variables and wandoo health change, linear regression analysis was performed. To determine if the changes in climate that occurred between the surveys were significant, paired Asymptotic Wilcoxon-Signed-Rank Tests were performed. For this, the changes in temperature and rainfall variables were compared between the 6-year average of 2002 and 2008, and to determine if the observed changes (i.e. increase or decrease) were significant compared with the long-term average, comparisons were made between the 6-year average of 2008 and the long-term 30-year average. Additionally, effect size (*r* = *z*/√2*n*) of the observed changes was calculated and interpreted following Cohen (1988), with *z* being the statistic given for the Wilcoxon test, and *n* the number of observations used (=115). To investigate the relationships between the continuous climatic variables and wandoo health change, linear regression analysis was performed. Variables that showed significant relationships with changes in wandoo health were used to explore first and second order variable combinations (i.e. interactions). Individual variables were combined based on the level of correlation (*r* > 0.8). The relationship between these interaction variables and wandoo health change were explored with linear regression.

Additionally, to answer the question of what combination of variables explained wandoo health change best; multiple linear regression models were constructed using all continuous variables and interaction variables. First, forward-stepwise multiple regression analysis was performed to explore potential variable combinations. Second, hierarchical multiple regression was performed, using variable combinations based on knowledge of the level of correlation between the variables (only variables with correlation level *r* < 0.2 were included). Final model selection was based on: (1) significance of individual variables adding to the model (*P* < 0.05); (2) homogeneity of variance (i.e. random residual distribution) and normality of the data (i.e. linear distribution in Normal Q-Q plot); and (3) the overall model fit (*P* < 0.05 and Adjusted *R*^2^).

## Results

Based on the latitude and longitude coordinates recorded in this study and available in the NatureMap database (DEC 2012), an outline (minimum bounding geometry) was created including all recorded point locations of wandoo in SWWA. This outline represents the extent of the area where the species can occur and was calculated to be ∼86,500 km^2^ (Fig. 1). The survey that was undertaken covered the larger part of this area, with the individual plots showing a good representation of the climatic range of wandoo (Fig. 1).

The average long-term annual rainfall (1976–2005) across all survey plots ranged between 339 and 859 mm, with spring averages of between 67 and 181 mm, summer: between 39 and 76 mm, autumn: between 75 and 167 mm, and winter: between 141 and 468 mm. Average long-term annual temperatures ranged between 13.5 and 18.4°C, with spring: between 12.2 and 16.8°C, summer: between 17.9 and 24.5°C, autumn: between 14.8 and 19.6°C, and winter: between 9.3 and 13.1°C.

Of the 115 plots that were included in the analyses, 66 were found to have declined in overall crown health between 2002 and 2008. Twenty plots were found to be stable and 29 plots had improved in health. Across all plots, canopy health change ranged between –38% and +21%.

### Fragment metrics

The woodland fragments including the survey plots were highly variable in perimeter and size (i.e. area), ranging between 0.2 and 115.6 km, and 0.0025 and 285.6 km^2^, respectively, with one large fragment of 424.2 km and 1,154.0 km^2^ in size. Inspection of the dataset used revealed that this large fragment was poorly digitized, ignoring clear separating boundaries such as roads and tracks. Therefore, all twelve plots situated within this single large fragment were removed prior to analysis. A clear positive relationship was found between canopy health change and both stand area and perimeter, with small fragments with long edges showing the largest declines (Linear Regression: *F* = 18.05; 18.86, df = 101, *P* < 0.001, Adjusted *R*^2^ = 0.143; 0.149, respectively). Additionally, the fragment shape index (FRAC) was found to be positively related to crown health change, where wandoo in more complex fragments (i.e. with a high perimeter/area ratio) showed the strongest declines (*F* = 7.825, df = 113, *P* = 0.006, Adjusted *R*^2^ = 0.057).

### Topography

Slope of the plots ranged from 0.2° to 14.9° with a mean of 2.5°. The distribution was highly skewed toward small slope values (skewness >2.9). After removing outlying plots situated on slopes >6° (*n* = 6), and applying log10 data transformation, no relationship was found between slope and canopy health change of wandoo (*F* = 0.963, *P* = 0.329, df = 107). Plots were found at elevations ranging from 22 to 402 m with an average elevation of 278 m. No relationship was found between elevation and canopy health change (*F* = 0.078, *P* = 0.781, df = 113 for all following). Fifty plots were found on north-facing slopes and 65 were found on south-facing slopes. No differences were found for canopy health change with north- or south-facing aspect (Welch two-sample *t*-test: *t* = 0.593, *P* = 0.554). Thus, topography was found to be unrelated to the observed canopy health change of wandoo.

### Soil characteristics

The likely presence and influence of shallow soils (i.e. distance to a rock layer in the soil profile, <2 m) and saline properties were generally low for the survey plots (average: 24.6% and 7.7%, range: 0.0–46.0 and 0.0–36.4, respectively), and were both found to be unrelated to canopy health change (*F* = 0.573, *P* = 0.450; *F* = 0.205, *P* = 0.651, respectively). These results indicate that the observed canopy health change of wandoo at the survey plots was not directly related to these soil characteristics.

### Climate changes

**Figure 2:**
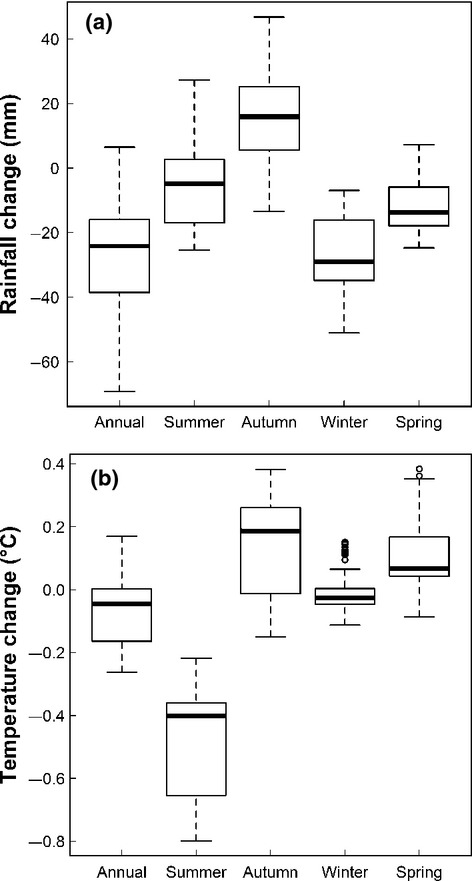
Changes in rainfall (a) and temperature (b) for all wandoo plots (*n* = 115) calculated as average 2008 (2002–2007) minus average 2002 (1996–2001). The box plots indicate the median and range. Summer: Dec–Feb, Autumn: Mar–May, Winter: Jun–Aug, Spring: Sep–Nov.

The changes in the climate variables that occurred over the 6 years between the surveys are displayed in Fig. 2. For the changes in rainfall, all changes were significant showing an overall and seasonal significant decrease in rainfall between surveys and compared with the 30-year average (Asymptotic Wilcoxon-Signed-Rank Test: *z* = −3.595 to −9.307, *P* = <0.001 and *z* = −4.521 to −9.277, *P* = <0.001, respectively). Autumn was an exception showing a significant increase in rainfall between the surveys and compared with the long-term average (*z* = 8.386, *P* = <0.001 and *z* = 5.582, *P* = <0.001, respectively). Calculations of the effect size indicating the relative weight of the changes revealed that the decreases were most prominent for winter (*r* = 0.61) and annual rainfall (*r* = 0.60) (following Cohen 1988), with all plots experiencing a decrease in winter rainfall (July–August) between 2% and 24% (Fig. 2a). The changes in temperature between the 6-year averages for 2002 and 2008 were all significant; however, comparisons with the long-term average found no change in average annual temperature (*z* = 1.462, *P* = 0.144), whereas winter temperatures showed a significant increase (*z* = 6.681, *P* = <0.001). The temperature increase found for spring and autumn was consistent with the long-term average (*z* = 7.452; 7.513, *P* = <0.001, and *z* = 9.307; 7.036, *P* = <0.001, respectively), and summer temperatures significantly dropped in all plots (*z* = −9.307; −9.307, *P* = <0.001). These changes were primarily driven by increases and decreases in maximum temperatures. Calculations of the effect size revealed that the temperature changes were most prominent for summer (*r* = 0.61), autumn (*r* = 0.50), and spring (*r* = 0.49), with all plots experiencing a significant drop in summer temperature (range: −0.80 to −0.22°C) (Fig. 2b). Compared with the long-term average rainfall and temperatures, all the observed climatic changes described above occurred at an equal relative magnitude across all plots.

### Tree health change and climate

**Figure 3:**
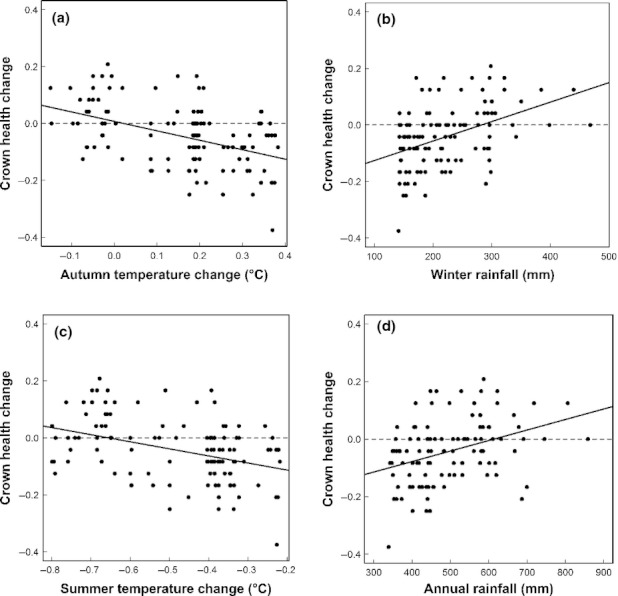
Relationships between crown health change and individual climate-related variables. Graph (a) and (c) indicate crown health declines in areas where autumn temperatures increased (a) or summer temperatures showed only little decrease (c) between 2002 and 2008. Graphs (b) and (d) indicate crown health declines predominantly in areas with low 30-year average winter (b) and/or annual (d) rainfall. For related statistics, see Table 2.

Several climate variables were significantly associated with the observed changes in wandoo crown health across the 115 plots. Decreases in crown health between the two surveys were most significantly associated with (1) areas that experienced an increase in temperature during autumn; (2) areas receiving low long-term average winter rainfall (i.e. eastern end of the wandoo range, see Fig. 1); (3) areas that experienced the smallest decrease in summer temperatures (i.e. where temperatures remained high); and (4) areas receiving low long-term average annual rainfall (see Fig. 3, Table 2). Interaction variables that showed the highest significant association with crown health change were “Autumn temperature change * Summer temperature change” and “Winter rainfall * Annual rainfall change” (Table 3). These interactions showed that wandoo mainly decreased in health: (1) where the combined summer and autumn temperatures increased between the surveys; and (2) where annual rainfall decreased in the low winter rainfall areas. Additionally, modeled average (30-year) summer soil moisture availability up to 1.5 m deep was found to be positively related to wandoo health change (*F* = 15.28, df = 113, *P* < 0.001, Adjusted *R*^2^ = 0.111).

**Table 2:** Climate-related relationships for the change in wandoo crown health across their geographic distribution

Linear regression model	Explanatory variables	*β*	F	df	*P*	*R*^2^	Adjusted *R*^2^
Y = *β*_0_+*β*_1_A	Autumn temperature change (°C)	−3.32E-01	28.46	113	<0.001	0.201	0.194
	*Intercept* (*β*_0_)	7.57E-03					
Y = *β*_0_+*β*_1_B	Winter rainfall (mm)	6.90E-04	26.59	113	<0.001	0.191	0.183
	*Intercept* (*β*_0_)	−1.95E-01					
Y = *β*_0_+*β*_1_C	Summer temperature change (°C)	−2.48E-01	20.07	113	<0.001	0.151	0.143
	*Intercept* (*β*_0_)	−1.62E-01					
Y = *β*_0_+*β*_1_D	Annual rainfall (mm)	3.65E-04	16.49	113	<0.001	0.127	0.120
	*Intercept* (*β*_0_)	−2.24E-01					

Y = Crown health change for wandoo between 2002 and 2008 as a fraction between −1 and +1.

**Table 3:** Multiple linear regression models including relevant interaction terms explaining the change of wandoo crown health between 2002 and 2008 across their geographic distribution

Multiple regression model	Explanatory variables	*β*	F	df	*P*	*R*^2^	Adjusted *R*^2^
Y = *β*_0_+*β*_1_A+*β*_2_BE	Autumn temperature change	−2.57E-01	17.46	112	<0.001	0.238	0.224
	Winter rainfall * Annual rainfall change	−5.09E-06					
	*Intercept* (*β*_0_)	−3.50E-02					
Y=*β*_0_+*β*_1_AC+*β*_2_BE	Autumn temp change * Summer temp change	6.17E-01	17.05	112	<0.001	0.233	0.220
	Winter rainfall * Annual rainfall change	−5.98E-06					
	*Intercept* (*β*_0_)	−4.94E-02					
Y=*β*_0_+*β*_1_C+*β*_2_BE	Summer temperature change	−1.75E-01	14.41	112	<0.001	0.205	0.190
	Winter rainfall * Annual rainfall change	−6.08E-06					
	*Intercept* (*β*_0_)	−1.64E-01					
Y = *β*_0_+*β*_1_AC	Autumn temp change * Summer temp change	8.04E-01	24.56	113	<0.001	0.179	0.171
	*Intercept* (*β*_0_)	−3.79E-03					
Y=*β*_0_+*β*_1_BE	Winter rainfall * Annual rainfall change	−8.93E-06	19.04	113	<0.001	0.144	0.137
	*Intercept* (*β*_0_)	−9.68E-02					

Y = Crown health change for wandoo between 2002 and 2008 as a fraction between −1 and +1. Capital letters in the model formula correspond with model variables from Table 2 with the addition of “E” representing “Annual rainfall change”. All significant variables were used in the exploration and selection of the models.

The best fitting multiple regression models describing crown health change were found to include climate-related variables only (Table 3). All other variables did not significantly add to the models. The best-fit model was found to include the variable representing the change in autumn temperature, and the variable representing the interaction between average (30-year) winter rainfall and the changes in annual rainfall that occurred at the survey plots between 2002 and 2008 (Table 3). Exchanging autumn temperature change with the variable representing the interaction between autumn and summer temperature change produced an equally well-performing model. Exchanging the interaction between winter rainfall and annual rainfall change with the interaction between annual rainfall and annual rainfall change also produced an equally well-performing model (*F* = 17.10, df = 113, *P* < 0.001, Adjusted *R*^*2*^ = 0.220).

Altogether, these analyses showed that crown health mainly decreased in winter (or annual) dry areas (Fig. 3b, d), mainly influenced by (1) the decreases in annual/winter rainfall (Fig. 2a); (2) increases in autumn temperature (Fig. 3a); and (3) relatively low decreases in summer temperature (Fig. 3c). Wandoo therefore seems most sensitive to the changes in climate in the low rainfall zone of its range (Fig. 1).

## Discussion

This study contributes to our understanding of how an endemic *Eucalyptus* species responds to changes in climate in a highly fragmented landscape. It is novel in that it uses a large spatio-temporal approach by (1) measuring health of a tree species across its entire distribution; (2) using field data collected at two points in time; and (3) relating the changes in health to landscape and climate variables in a threatened Mediterranean ecoregion. This study also provides one of the first recorded pieces of evidence in support of bioclimatic modeling studies (Hughes et al. 1996; Klausmeyer and Shaw 2009), showing the negative response of an important tree species in SWWA to changes and shifts in climate.

The statistical models strongly suggest that average annual winter rainfall and recent shifts in climate (i.e. equal relative increases in autumn temperature and decreases in annual rainfall across the wandoo climate gradient) are associated with the health decline of wandoo (Table 3). The relatively low amount of explained variation by the models indicates that the declines are probably influenced by multiple interacting factors including pests and pathogens (Hooper and Sivasithamparam 2005), and episodic events such as droughts, heat waves, and fires. However, including these factors was beyond the scope of this study. Despite these and other limitations (i.e. relatively coarse resolution of climate datasets, and only using two points in time for our analysis), our findings correspond with previous research on climate and wandoo health. The historical long-term climate trends for SWWA show decreases in rainfall and increasing temperatures (Bates et al. 2008), and the observed health of wandoo has increasingly been declining (Hooper and Sivasithamparam 2005; Wandoo Recovery Group 2006; Gaynor 2008). This strongly suggests that the changes in climate have been negatively affecting wandoo health (Fig. 3b, d), particularly in the low rainfall zone of its distribution (Fig. 1). Equally, specific Mediterranean studies showed decreases in tree health with increased water deficits (Carnicer et al. 2011; Sánchez-Salguero et al. 2012), and decreased tree growth associated with less rainfall and increased temperatures in Spain (Vilà-Cabrera et al. 2011); reduced tree growth related to less rainfall on Greek islands (Sarris et al. 2007, 2011); and increasing temperatures affecting *Fagus sylvatica* (beech) growth at the southern edge of its range in Spain (Jump et al. 2006). Long-term increases in temperature and water deficits were further found to be the main driver for increased tree mortality rates in the boreal forests of Canada (Peng et al. 2011), in the Sierra Nevada of California (van Mantgem and Stephenson 2007), across the western United States including forests in the Mediterranean region (van Mantgem et al. 2009), and in Europe (Carnicer et al. 2011; Vilà-Cabrera et al. 2011), indicating the significance of our findings in relation to global observations (Allen et al. 2010).

The climatic changes in SWWA toward prolonged warmer conditions running into autumn, in combination with reduced rainfall, is likely resulting in more pronounced soil water deficits negatively affecting wandoo health. Where the magnitude of the climatic changes was equal across the wandoo range, physiological constraints are likely to make wandoo at the eastern dry (and warm) end of its range most susceptible to these changes, resulting in declining health. The high likelihood of a continuation of the observed climate change trends in SWWA (i.e. drying and warming) (CSIRO & BOM 2007) combined with our results suggests that unless wandoo is able to adapt phenotypically and/or genetically, it is likely to become less dominant or even disappear from its dry eastern range limit. Similarly, dramatic shifts have also been suggested by Hughes et al. (1996) in an earlier modeling study investigating bioclimatic change scenarios for *Eucalyptus* species across Australia. They predicted that the current climatic suitability for many species in SWWA would shift considerably or disappear all together under future climate change scenarios (Hughes et al. 1996), exacerbated by SWWA highly fragmented environment and natural boundaries (i.e. surrounding oceans and arid interior) (Fig. 1).

The recorded onset of the decline in wandoo coincided with significant declines and shifts in rainfall, and the commencement of increasing temperatures in the SWWA since the mid-1970s (Bates et al. 2008; Gaynor 2008). This corresponds with the climatic trends and tree health responses observed in Spain (Jump et al. 2006; Carnicer et al. 2011). Carnicer et al. (2011) found that since the second half of the 20th century, increasing water deficits as a function of temperature and rainfall were strongly related to decreased crown health condition of 16 tree species particularly in the drier part of the species' range. Jump et al. (2006) found that declines in *F. sylvatica* growth at its southern range edge commenced around 1975 and were related to increasing temperatures. Equally, wandoo has progressively shown phases of decline (and partial recovery) and mortality at a local scale since significant drying and warming occurred (Wandoo Recovery Group 2006; Gaynor 2008), but with our results now suggesting a more gradual continuing health decline across the drier part of its range. Apart from wandoo, other woody species in SWWA have increasingly shown phases of decline and mortality (Cai et al. 2010; Brouwers et al. 2012; Matusick et al. 2012), indicating that declines in tree health are becoming more prevalent in this region. The similarities in climate change projections specific for Mediterranean ecoregions (i.e. unique combination of drying and warming) (IPCC 2007b), and the similar decline responses in the Mediterranean forests of the Northern and Southern hemisphere (Jump et al. 2006; Sarris et al. 2007, 2011; Allen et al. 2010; Carnicer et al. 2011), indicate the likely generality of our findings.

Besides the apparent climate-health relationship, wandoo in forest fragments that were small and with a relative large perimeter/area ratio (i.e. complex-shaped) were generally found to be declining. Similarly, Barbeta et al. (2011) found that dominant mature *F. sylvatica* trees in forest fragments in Spain displayed more crown damage than trees in more continuous forest, indicating a potential negative fragmentation effect. In a recent review on the interactions between climate change and habitat loss effects, Mantyka-Pringle et al. (2012) concluded that in areas characterized by high maximum temperatures and where annual rainfall had decreased over time, native vegetation was most sensitive to the negative effects of habitat loss and fragmentation. Our results suggest that wandoo in the fragmented dry eastern end of its range (Fig. 1) is potentially impacted by this cumulative effect. Investigations of the combined effects of these and other interacting disturbance processes on the health of forests have been lacking, particularly in SWWA. Persistence of the global climate and environmental changes emphasizes the importance of understanding these interactions to generate necessary information for the development of appropriate conservation management strategies (Mantyka-Pringle et al. 2012).

Permanent and consistent tree health monitoring programs like those established in the United States (Stolte 2001; Bennett and Tkacz 2008) and Europe (as used and cited in Carnicer et al. 2011) will be of key importance to provide information related to the changes in forest ecosystems driven by climate change (Stolte 2001). Large-scale monitoring programs for multiple tree species are currently lacking across the unique Mediterranean ecoregion of SWWA. Since this ecoregion is of global importance due to its high biodiversity values (Hopper and Gioia 2004; Klausmeyer and Shaw 2009), knowledge of how future climate change impacts on species' ranges and persistence will be paramount for conservation management and planning. Therefore, establishment and continuation of large spatio-temporal scale monitoring and research programs in Mediterranean ecoregions should be encouraged. The information that is generated from these efforts will be highly valuable for providing the essential baseline spatio-temporal information on the wider changes that are likely to occur in SWWA and Mediterranean ecoregions around the world (Carnicer et al. 2011). Management of these unique environments can only then be tailored appropriately (Millar et al. 2007).
